# Clinical characteristics, management, and outcomes of polytraumatized children: a retrospective cohort study

**DOI:** 10.1186/s12245-026-01371-2

**Published:** 2026-09-10

**Authors:** Lisa Fiona Roder, Soufian Ben Amar, Alberto Alfieri Zellner, Jonas Roos, Johanna Thiele, Lioba Bürvenich, Kristian Welle, Christian Prangenberg

**Affiliations:** Department of Orthopedics and Trauma Surgery of the University Hospital Bonn, Bonn, Germany

**Keywords:** Pediatric trauma, Polytrauma, Subdural hematoma, Skull fracture, ICU, Outcomes

## Abstract

**Introduction:**

Pediatric polytrauma remains a major cause of morbidity and mortality worldwide. Owing to age-specific anatomical and physiological characteristics, children are particularly vulnerable to traumatic brain injury (TBI), which frequently represents the most severe component of polytrauma. This study aimed to evaluate injury mechanisms, injury patterns, treatment strategies, complications, and outcomes in pediatric polytrauma patients treated at a tertiary trauma center.

**Methods:**

A retrospective single-center cohort study was conducted including pediatric patients (< 18 years) with polytrauma, meaning with an Injury Severity Score (ISS) ≥ 16, treated at the University Hospital Bonn between 2023 and 2025. Demographic data, injury mechanisms, injury patterns, ISS, Maximum Abbreviated Injury Scale (MAIS) scores, surgical interventions, intensive care unit (ICU) treatment, complications, and discharge outcomes were analyzed. Correlations between head MAIS, ISS, age, and length of hospital stay were assessed using Spearman’s rank correlation coefficient, while differences in ISS between injury mechanisms were evaluated using the Kruskal–Wallis test.

**Results:**

Thirty-two pediatric patients were included. The median age was 13.5 years (range 2–17 years), and 50% were female. Traffic accidents were the predominant mechanism of injury (75%), followed by falls from heights greater than 3 m (12.5%). Head trauma was the most common injury pattern, including skull fractures (34.4%), subarachnoid hemorrhage (28.1%), subdural hematoma (25.0%), and epidural hematoma (9.4%). Thoracic, abdominal, pelvic, and extremity injuries were also frequently observed. Fifteen patients (46.9%) required surgical intervention. The median ICU stay was 3 days (range 0–69 days). Complications occurred in five patients (15.6%) and included meningitis, aseptic bone necrosis, epidural rebleeding, soft tissue infection, and pulmonary complications. No in-hospital mortality was observed. Most patients (84.4%) were discharged directly home, while 6.3% required inpatient rehabilitation and 9.4% were transferred to another hospital. No significant correlations were found between head MAIS and length of hospital stay (ρ = 0.28, *p* = 0.127), head MAIS and ISS (ρ = 0.07, *p* = 0.714), ISS and hospitalization duration (ρ = 0.10, *p* = 0.581), or age and head MAIS (ρ = 0.03, *p* = 0.883).

**Conclusion:**

TBI represented the predominant injury burden and was associated with the most complex clinical courses. Despite the severity of injuries, outcomes were generally favorable, characterized by low complication rates, absence of in-hospital mortality, and a high proportion of patients discharged home. These findings emphasize the importance of specialized multidisciplinary trauma care, neurosurgical expertise, and pediatric intensive care management in severely injured children. Larger multicenter studies with long-term follow-up are warranted to further identify predictors of outcome and optimize pediatric polytrauma care.

## Introduction

Pediatric polytrauma remains one of the leading causes of morbidity and mortality in children and adolescents worldwide [1, 2]. In contrast to adults, pediatric trauma patients exhibit distinct anatomical and physiological characteristics that substantially influence injury patterns, clinical presentation, and therapeutic management. The proportionally larger head size, reduced cranial bone thickness, and ongoing neurodevelopment render children particularly susceptible to TBI, while the increased elasticity of the thoracic cage may conceal severe intrathoracic injuries despite minimal external signs of trauma [3–5].

Over recent decades, advances in prehospital care, trauma systems, neurosurgical techniques, and pediatric intensive care management have significantly improved survival rates in severely injured children [2, 6, 7]. Nevertheless, pediatric polytrauma continues to be associated with considerable mortality, prolonged hospitalization, and long-term neurological and functional impairment [8, 9]. Severe TBI remains a major determinant of adverse outcomes, frequently requiring intensive multidisciplinary management and prolonged rehabilitation [5, 10]. In addition, associated thoracic, abdominal, and orthopedic injuries contribute substantially to morbidity and complexity of care [11–15].

Previous studies and trauma registry analyses have identified traffic accidents and high-energy falls as the predominant mechanisms of severe pediatric trauma [1, 16]. Furthermore, injury severity scores such as the Injury Severity Score (ISS) and the Maximum Abbreviated Injury Scale (MAIS) have become established tools for trauma assessment and prognostic evaluation [17]. However, detailed data regarding the relationship between regional injury severity, intensive care treatment, complications, and functional outcomes in pediatric polytrauma patients remain limited, particularly in single-center cohorts from tertiary trauma centers in Germany [6, 18].

The present study aims to provide a comprehensive analysis of pediatric polytrauma patients treated at the University Hospital Bonn over a 3-year period. Special emphasis was placed on injury mechanisms, TBI patterns, intensive care treatment, complications, and discharge outcomes. Furthermore, potential associations between ISS, head MAIS, age, and duration of hospitalization were evaluated in order to identify clinically relevant factors associated with adverse outcomes and prolonged recovery in severely injured children [8, 17, 18].

## Methods

This study was designed as a retrospective single-center cohort analysis of pediatric patients younger than 18 years of age who were admitted to the Department of Orthopedics and Trauma Surgery at the University Hospital Bonn between 2023 and 2025. Patients were eligible for inclusion if they fulfilled the criteria for polytrauma, defined as multiple severe injuries and/or an Injury Severity Score (ISS) ≥ 16. Patients with isolated injuries or incomplete medical records were excluded from the analysis. Clinical and demographic data were extracted retrospectively from the institutional electronic medical record system. Collected demographic variables included age and sex. Mechanisms of injury were categorized into predefined groups, including traffic accidents, falls from heights greater than three meters, trampoline-related injuries, horse-related injuries, suicide attempts, and penetrating trauma. Injury patterns were systematically documented and included cranial, thoracic, abdominal, pelvic, and extremity injuries. Traumatic brain injuries were further classified according to specific diagnoses, including subarachnoid hemorrhage (SAH), subdural hematoma (SDH), epidural hematoma (EDH), and skull fractures. Injury severity was assessed using the Injury Severity Score (ISS) and the Maximum Abbreviated Injury Scale (MAIS), including regional MAIS scores and head MAIS. Therapeutic interventions, including neurosurgical and orthopedic surgical procedures, were recorded. Furthermore, intensive care unit (ICU) treatment, duration of ICU stay, length of hospital stay, discharge destination, and in-hospital complications were documented. Reported complications included infectious complications, meningitis, aseptic bone necrosis, epidural rebleeding, soft tissue infection, prolonged mechanical ventilation, and pulmonary complications. Discharge destination was categorized as discharge home, transfer to an inpatient rehabilitation facility, or transfer to an external hospital for continued treatment. Statistical analyses were performed using SPSS Statistics version 28 (IBM Corp., Armonk, NY, USA). Continuous variables were expressed as median and range or interquartile range (IQR), whereas categorical variables were reported as absolute numbers and percentages. Correlation analyses between head MAIS, ISS, age, and length of hospital stay were performed using Spearman’s rank correlation coefficient. Differences in ISS between injury mechanisms were assessed using the Kruskal–Wallis test. Statistical significance was defined as a two-sided p-value < 0.05. According to the Ethics Committee of the University Hospital Bonn, the retrospective analysis of data extracted from patient records did not require explicit written informed consent because no additional data were collected after completion of treatment (approval number 406/17). All patient data were anonymized prior to analysis, and the study was conducted in accordance with the principles of the Declaration of Helsinki.

## Results

A total of 32 pediatric patients met the inclusion criteria for this study. The median age was 13.5 years (range: 2–17 years). The cohort consisted of 16 males (50%) and 16 females (50%) with a median ISS of 27.

**Fig. 1:**
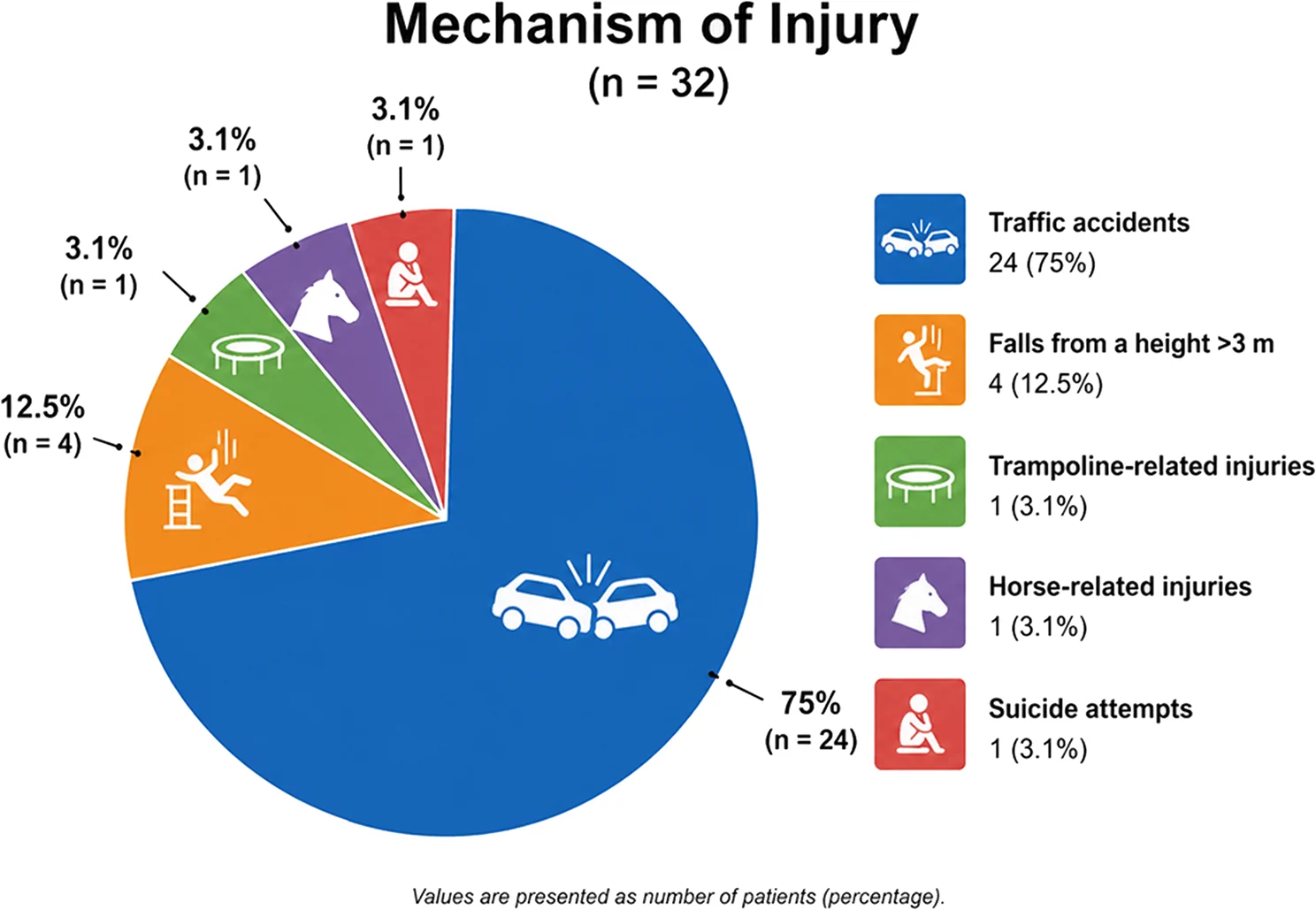
Distribution of mechanisms of injury within the study cohort (*n* = 32). Traffic accidents represented the most common mechanism of injury, accounting for 75% of all cases, followed by falls from heights greater than 3 m (12.5%). Trampoline-related injuries, horse-related injuries, and suicide attempts each accounted for 3.1% of cases. Values are presented as absolute numbers and percentages

Traffic accidents represented the predominant mechanism of injury, accounting for 24 patients (75%). Falls from heights greater than 3 m occurred in 4 patients (12.5%). Horse-related trauma, trampoline-related trauma, suicide attempts, and penetrating trauma due to a knife injury were each observed in 1 patient (3.1%) (Fig. 1).

Head injuries were the most common injury pattern. Subarachnoid hemorrhage (SAH) was documented in 9 patients (28.1%), subdural hematoma (SDH) in 8 patients (25.0%), and epidural hematoma (EDH) in 3 patients (9.4%). Skull fractures were present in 11 patients (34.4%). Thoracic injuries, including pneumothorax, pulmonary contusions, and rib fractures, were frequently observed, while abdominal injuries mainly consisted of splenic and hepatic trauma. Extremity and pelvic fractures were also common. The detailed distribution of injuries is summarized in Fig. 1.

Fifteen patients (46.9%) required operative treatment. The median duration of intensive care unit (ICU) stay was 3 days, ranging from 0 to 69 days.

Complications occurred in 5 patients (15.6%). Reported complications included aseptic bone necrosis following cranioplasty, meningitis requiring prolonged mechanical ventilation, soft tissue infection, epidural rebleeding, and persistent pulmonary complications with intrapulmonary hematoma, prolonged pneumothorax, and fistula formation.

No patient died during the hospital stay. At discharge, 27 patients (84.4%) were discharged directly home, 2 patients (6.3%) were transferred to an inpatient rehabilitation facility, and 3 patients (9.4%) required transfer to an external hospital for further treatment.

**Fig. 2:**
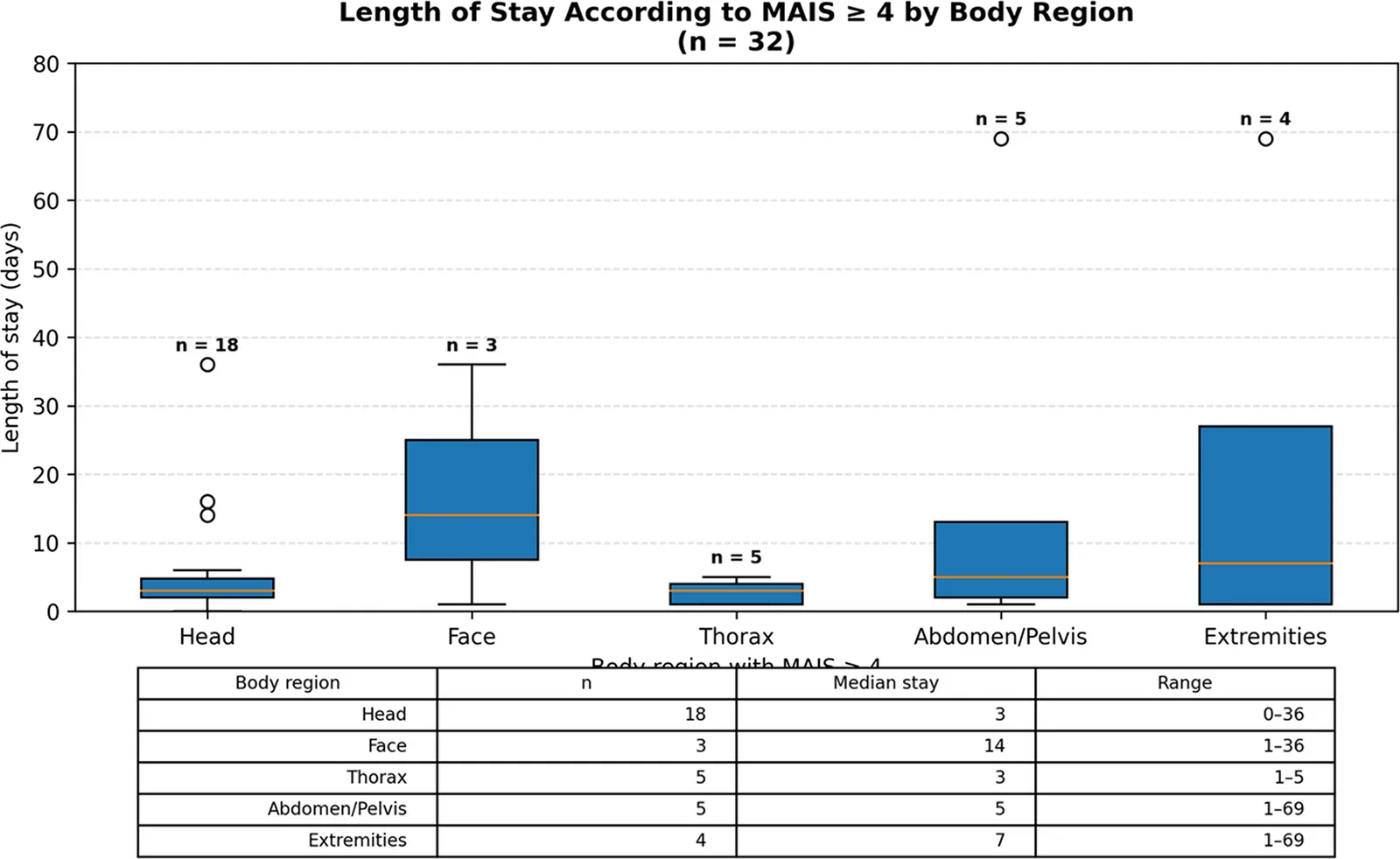
Length of hospital stay according to body region with severe injury (MAIS ≥ 4) in pediatric polytrauma patients (*n* = 32). Boxplots illustrate the distribution of hospital length of stay for patients with severe injuries (MAIS ≥ 4) in different body regions. Head injuries were the most common severe injury pattern (*n* = 18), whereas severe facial, thoracic, abdominal/pelvic, and extremity injuries occurred less frequently. The longest and most variable hospital stays were observed among patients with severe abdominal/pelvic and extremity injuries, with maximum lengths of stay reaching 69 days. Median length of stay was 3 days for head injuries, 14 days for facial injuries, 3 days for thoracic injuries, 5 days for abdominal/pelvic injuries, and 7 days for extremity injuries. The table summarizes the number of patients, median hospital stay, and range for each injury region. Values are presented as median and range

Correlation analyses using Spearman’s rank test did not demonstrate a statistically significant association between head MAIS and length of hospital stay (ρ = 0.28, *p* = 0.127), suggesting that the severity of cranial injury alone was not predictive of prolonged hospitalization in this cohort. Similarly, no significant correlation was identified between head MAIS and overall injury severity as measured by the ISS (ρ = 0.07, *p* = 0.714). Fig. 2.

**Fig. 3:**
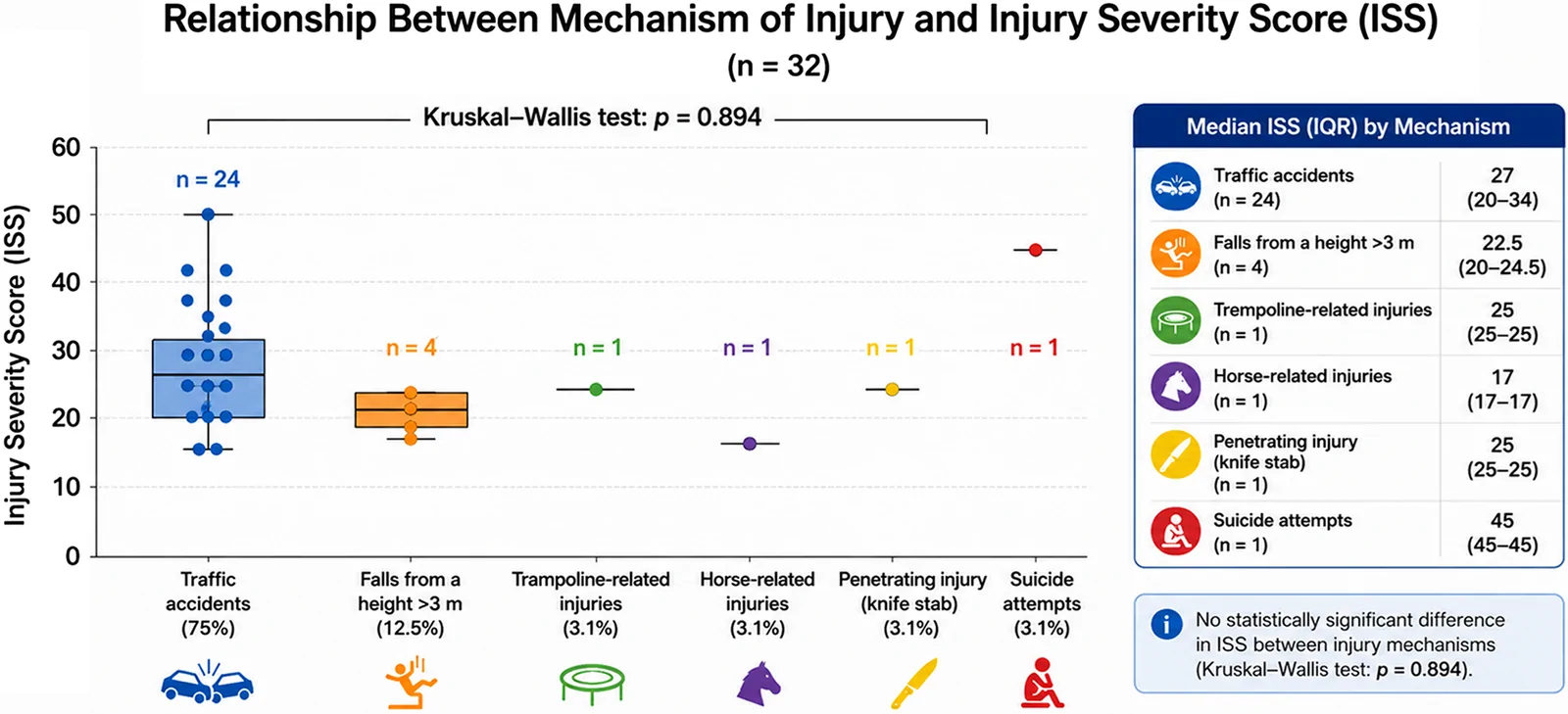
Relationship between mechanism of injury and Injury Severity Score (ISS) in pediatric polytrauma patients (*n* = 32). Boxplots illustrate the distribution of ISS values according to injury mechanism. Traffic accidents were the predominant mechanism of injury (75%) and demonstrated the widest range of ISS values, reflecting the heterogeneous severity of traffic-related trauma. Falls from heights greater than 3 m accounted for 12.5% of cases, while trampoline-related injuries, horse-related injuries, penetrating injuries (knife stab), and suicide attempts each represented 3.1% of the cohort. Median ISS values and interquartile ranges are displayed for each injury mechanism. No statistically significant differences in ISS were observed between injury mechanisms (Kruskal–Wallis test, *p* = 0.894). Values are presented as median (IQR)

**Fig. 4:**
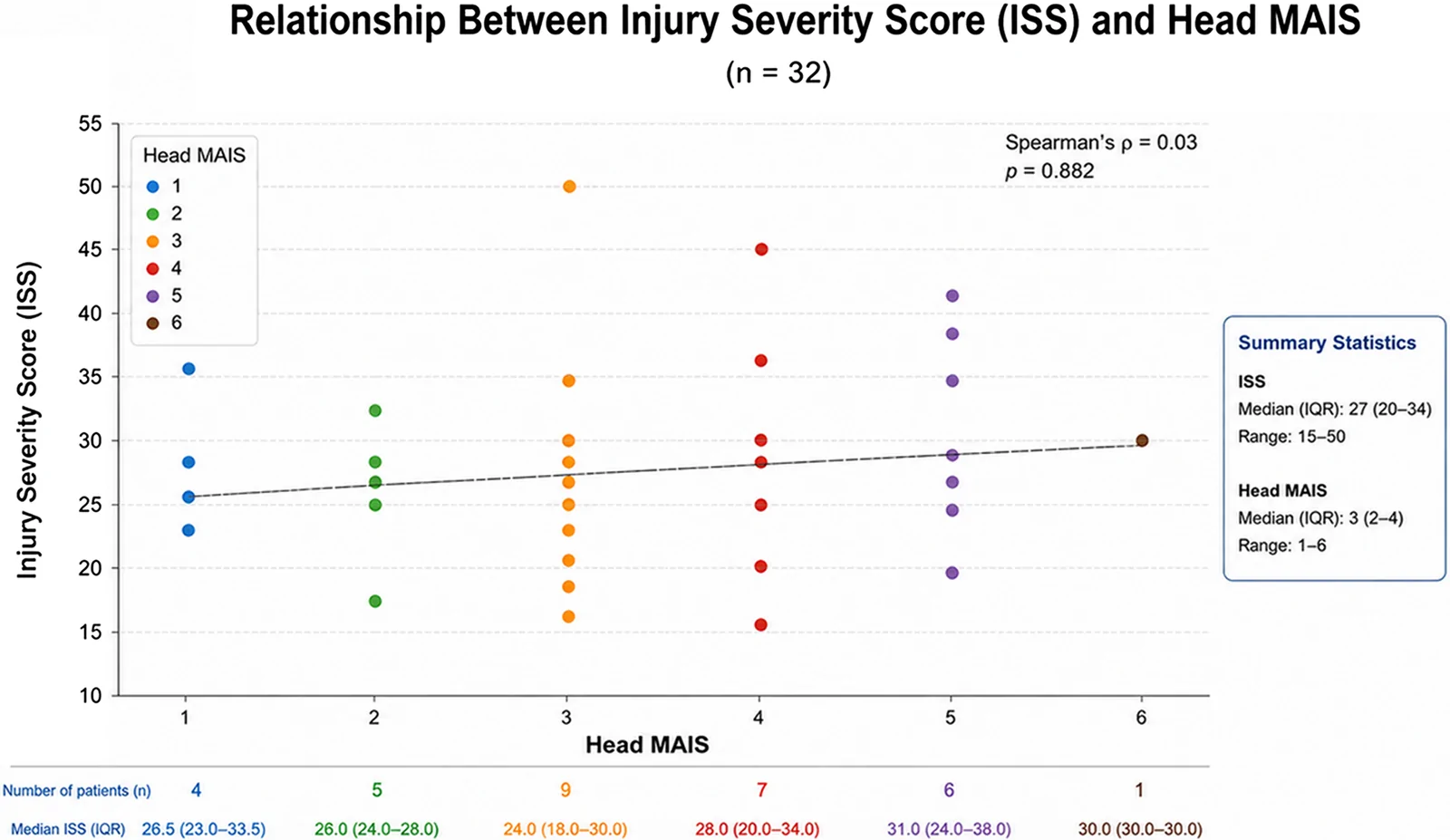
Relationship between Injury Severity Score (ISS) and Head Maximum Abbreviated Injury Scale (MAIS) in pediatric polytrauma patients (*n* = 32). Scatter plot illustrating ISS values according to Head MAIS category. Each dot represents an individual patient, and the dashed line indicates the overall trend between regional head injury severity and overall trauma burden. Head MAIS scores ranged from 1 to 6, with a median of 3 (IQR 2–4), while the median ISS was 27 (IQR 20–34). No significant correlation was observed between Head MAIS and ISS (Spearman’s ρ = 0.07, *p* = 0.714), indicating that the severity of cranial injury was not significantly associated with overall injury severity in this cohort. The number of patients and median ISS values (IQR) for each Head MAIS category are displayed below the graph. Values are presented as median (IQR)

In addition, ISS did not show a statistically significant relationship with duration of hospitalization (ρ = 0.10, *p* = 0.581). Patient age was likewise not significantly associated with the severity of head injury (ρ = 0.03, *p* = 0.883), indicating that severe cranial trauma occurred across all pediatric age groups without a clear age-dependent distribution. Figs. 3 and 4.

Although statistical significance could not be demonstrated, severe TBI remained clinically associated with the highest burden of complications and the most complex clinical courses. Patients with high head MAIS scores more frequently required intensive care treatment, operative intervention, and prolonged hospitalization.

## Discussion

This study characterizes the injury patterns, management, and short-term outcomes of pediatric polytrauma patients treated at a tertiary trauma center over a three-year period. The cohort was characterized by a predominance of traffic-related injuries and cranial trauma, considerable injury severity, and frequent requirements for operative and intensive care treatment. Despite this substantial injury burden, short-term outcomes were generally favorable. These findings should, however, be interpreted in the context of the small sample size and retrospective single-center design. They still emphasize the importance of specialized multidisciplinary trauma care and support the central role of neurosurgical expertise in the management of severely injured children [7, 19].

Head trauma represented the most frequent injury pattern in our cohort, with subarachnoid hemorrhages, subdural hematomas, and skull fractures occurring in a substantial proportion of patients. TBI is widely recognized as a major contributor to morbidity in pediatric polytrauma and constituted the predominant injury burden in the present study. Although no statistically significant association between head injury severity and clinical outcome parameters could be demonstrated, patients with severe traumatic brain injuries experienced the majority of documented complications, including infectious complications, prolonged mechanical ventilation, and persistent neurological sequelae. These findings support the clinical relevance of severe head trauma as an important determinant of complex treatment courses in pediatric polytrauma [5, 20]. Pediatric head trauma differs substantially from adult trauma due to the unique anatomical and physiological characteristics of the developing skull and brain. The greater cranial compliance, ongoing neurodevelopment, and age-dependent biomechanical properties influence both injury patterns and clinical presentation, making TBI a particularly important consideration in the management of severely injured children [3, 4]. While statistical analyses did not reveal significant associations between head MAIS and clinical outcome measures, severe TBI was frequently associated with complex treatment courses requiring intensive care and neurosurgical management. Given the predominance of cranial injuries in our cohort, these findings reinforce the central role of TBI in pediatric polytrauma care. Early neurosurgical intervention has been shown to improve neurological recovery and functional outcomes in children with severe TBI and therefore remains a cornerstone of treatment [10]. Given the high prevalence of TBIs in our cohort, the prevention of secondary brain injury remains a key therapeutic objective. Elevated intracranial pressure and other secondary injury mechanisms have been shown to contribute to unfavorable neurological outcomes in pediatric TBI, emphasizing the importance of specialized neurocritical care and early intervention in severely injured children [21].

Traffic accidents were by far the most common mechanism of injury in our cohort, accounting for three-quarters of all cases. Falls from heights greater than three meters represented the second most frequent injury mechanism, whereas other causes were only observed sporadically. These findings underline the major contribution of high-energy trauma to pediatric polytrauma and are in accordance with previous reports demonstrating that road traffic accidents remain a leading cause of severe injury in children and adolescents [1, 16, 22, 23]. The mechanism of injury differed descriptively across age groups. Among children aged 0–3 years, falls represented the most frequent mechanism of injury (66.7%), whereas traffic accidents became increasingly predominant with advancing age, accounting for 66.7% of injuries among children aged 4–10 years and 84.2% among patients aged 11–18 years. However, the association between age group and mechanism of injury did not reach statistical significance. Given the small number of patients, particularly in the youngest age group, these findings should be interpreted as exploratory.

While injury severity varied across trauma mechanisms, traffic accidents were associated with the highest number of severe injuries and the broadest ISS distribution. The median ISS among traffic accident victims was 27, compared with 22 among patients sustaining falls from heights greater than 3 m. However, no statistically significant association between injury mechanism and ISS could be demonstrated within this cohort [17]. While the Maximum Abbreviated Injury Scale (MAIS) provides a standardized assessment of regional injury severity and is widely used to characterize severe traumatic injuries, no significant associations between head MAIS and clinical outcome parameters were observed in our cohort [24].

Thoracic, abdominal, and extremity injuries occurred less frequently than cranial trauma but nevertheless contributed substantially to the overall injury burden of pediatric polytrauma patients. Thoracic injuries included pneumothorax, pulmonary contusions, hemothorax, and rib fractures, while abdominal trauma predominantly involved splenic and hepatic injuries. Severe extremity and pelvic injuries were also observed in several patients and frequently required operative management. Consistent with previous reports, thoracic trauma may be associated with respiratory compromise and the need for intensive care treatment in severely injured children [14, 15]. In our cohort, severe abdominal and extremity injuries were present in a limited number of patients, reflecting the heterogeneous injury patterns encountered in high-energy trauma. Although no statistically significant associations between regional injury severity and clinical outcome parameters were identified, these injuries often necessitated multidisciplinary treatment strategies involving pediatric surgeons, orthopedic surgeons, and intensive care specialists. Previous studies have demonstrated that many pediatric abdominal injuries can be managed selectively depending on hemodynamic stability, thereby avoiding unnecessary operative intervention [12]. Operative treatment was required in nearly half of all patients, highlighting the complexity of injury management in this population. Neurosurgical procedures constituted a substantial proportion of surgical interventions, reflecting the predominance of TBI within the cohort, while orthopedic stabilization was required in patients with severe fractures. Early definitive fracture management has been associated with reduced systemic stress and improved recovery in severely injured patients, emphasizing the importance of coordinated multidisciplinary trauma care [13].

The duration of intensive care treatment varied considerably within the cohort. While the median ICU stay was relatively short at 3 days, several patients required prolonged critical care management, with the longest ICU stay reaching 69 days. However, such a prolonged stay in the intensive care unit appears to be uncommon in pediatric and adolescent patients, as also suggested by comparison with the other available data. In our cohort, this extended ICU stay was attributable to the need for multiple complex surgical procedures and prolonged psychiatric care. Those findings illustrate the substantial heterogeneity in injury severity and clinical complexity among pediatric polytrauma patients. Previous studies have reported that prolonged ICU treatment in severely injured children is associated with an increased risk of complications and less favorable functional outcomes [18]. Complications were documented in 5 patients (15.6%) and included aseptic bone necrosis following cranioplasty, meningitis with prolonged mechanical ventilation, soft tissue infection, epidural rebleeding, and persistent pulmonary complications with intrapulmonary hematoma, prolonged pneumothorax, and fistula formation. Notably, several of these complications occurred in patients requiring extended intensive care treatment, emphasizing the challenges associated with the management of severe pediatric trauma. Infectious complications remain a particular concern during prolonged pediatric intensive care stays [25].

Direct comparison with previously published pediatric trauma cohorts is challenging because reported complication rates vary considerably depending on injury severity, study population, follow-up period, and the definition and ascertainment of complications. In a large multicenter analysis of more than 62,000 pediatric trauma patients, an overall complication rate of approximately 4% was reported, although this cohort included a substantially broader spectrum of injury severity and is therefore not directly comparable to our population of severely injured patients [26]. In contrast, earlier studies of selected pediatric polytrauma populations have reported considerably higher complication rates, with complications occurring in approximately one-third of patients.

Against this heterogeneous background, the complication rate of 15.6% observed in our cohort appears to fall within the broad range reported for pediatric trauma populations rather than being distinctly low or high. Given the median ISS of 27, the relatively limited number of documented complications may appear favorable; however, differences in complication definitions and reporting preclude direct benchmarking. In addition, the retrospective design of the present study may have resulted in underreporting of minor or transient complications that were not consistently documented in the medical records.

The observed complication rate should therefore be interpreted descriptively rather than as evidence of superior outcomes compared with previously published cohorts. Larger studies using standardized definitions of trauma-related complications and comparable injury-severity criteria are required to allow meaningful comparisons between pediatric polytrauma populations.

Despite the occurrence of these adverse events, no in-hospital mortality was observed, and the majority of patients were discharged directly home. These findings suggest that specialized multidisciplinary trauma care can achieve favorable outcomes even in severely injured pediatric patients, although prolonged ICU treatment and complications continue to represent important determinants of resource utilization and recovery [8].

Functional outcomes at discharge were generally favorable. The majority of patients (84.4%) were discharged directly to their home environment, while only 6.3% required transfer to an inpatient rehabilitation facility and 9.4% were transferred to an external hospital for continued treatment. These findings suggest that despite the severity of injuries and the frequent need for intensive care and surgical interventions, most patients achieved sufficient recovery to allow discharge home.

Nevertheless, a subset of patients required ongoing specialized care after acute hospitalization, highlighting the potential long-term burden associated with severe pediatric trauma. Rehabilitation following pediatric polytrauma plays a crucial role in restoring functional independence, supporting neurocognitive recovery, and facilitating reintegration into daily life and school activities [9].

Notably, no in-hospital mortality was observed in the present cohort. This favorable survival outcome may reflect advances in modern trauma systems, intensive care management, and the availability of specialized multidisciplinary treatment pathways for severely injured children [5, 18]. Previous studies have demonstrated improved outcomes and survival rates for pediatric trauma patients treated in specialized pediatric trauma centers, emphasizing the importance of centralized expertise in the management of severe childhood injuries [6, 7]. The findings appear favorable compared with previously published pediatric trauma cohorts with an ISS ≥ 16, in which mortality rates of approximately 5–15% have been reported. However, given the small sample size of the present cohort, the observed mortality rate should be interpreted cautiously and should not be considered evidence of superior survival compared with other pediatric trauma populations.

Several factors may have contributed to the absence of mortality. First, the organization and quality of care within a well-established regional trauma system, including rapid prehospital management, interdisciplinary treatment, and access to specialized pediatric intensive care and surgical expertise, may have contributed to favorable outcomes [27]. However, the present retrospective study was not designed to evaluate the effect of trauma system performance on mortality, and a causal relationship cannot be established. Furthermore, referral and transfer patterns may have introduced selection bias. Particularly severely injured children who died at the scene, during initial stabilization, or before transfer to our center would not have been captured by the present hospital-based cohort, potentially resulting in an underestimation of overall trauma-related mortality.

Finally, the distribution and anatomical pattern of injuries should be considered in addition to the overall ISS when interpreting mortality. Although the median ISS indicates substantial injury severity, ISS alone does not fully reflect the immediate physiological consequences or lethality of a specific injury pattern. A considerable proportion of patients may therefore have sustained anatomically severe injuries without injuries that were immediately life-threatening under appropriate treatment. Together with the limited cohort size, these characteristics may partly explain the observed 0% mortality. Larger multicenter studies including prehospital deaths, primary admissions, and interhospital transfers would be required to determine whether the low mortality observed in our cohort reflects characteristics of the study population, referral patterns, trauma system performance, or a combination of these factors.

An additional finding of the present study was the equal distribution of male and female patients within the study cohort. This differs from the male predominance commonly reported in pediatric trauma populations, in which approximately 60–70% of patients are typically male [28]. The balanced sex distribution observed in our cohort should therefore be noted as a characteristic of the present study population and may limit the direct comparability of our findings with larger pediatric polytrauma cohorts.

Several factors may have contributed to this finding. Most importantly, the relatively small sample size of 32 patients makes the observed sex distribution particularly susceptible to random variation. In addition, regional differences in trauma mechanisms, recreational activities, traffic exposure, and referral patterns may potentially influence the demographic composition of pediatric trauma populations. However, the present study was not designed to investigate regional or sex-specific differences in injury epidemiology, and no conclusions regarding the underlying causes of the balanced distribution can therefore be drawn from our data.

The equal representation of both sexes may nevertheless provide a useful perspective on severely injured pediatric and adolescent patients that differs from the predominantly male cohorts described in previous studies. At the same time, this finding emphasizes the need for cautious interpretation and external validation. Larger multicenter studies are required to determine whether the balanced sex distribution observed in our cohort represents a regional epidemiological characteristic or, more likely, reflects variation associated with the limited sample size.

Several limitations of this study should be acknowledged. First, the retrospective single-center design may limit the generalizability of the findings to other institutions and healthcare systems. Second, the relatively small cohort size of 32 patients reduced the statistical power of the analyses, particularly for subgroup comparisons and less common injury mechanisms such as horse-related trauma, trampoline injuries, suicide attempts, and penetrating injuries. Given the retrospective and exploratory nature of the study and the limited number of eligible pediatric trauma patients during the study period, no a priori sample size or power calculation was performed. Consequently, the statistical analyses should primarily be regarded as exploratory rather than confirmatory, and the absence of statistically significant associations should be interpreted with appropriate caution.

In particular, none of the four Spearman correlation analyses or the Kruskal–Wallis test demonstrated statistically significant associations or group differences. However, these non-significant findings cannot be interpreted as evidence for the absence of clinically relevant relationships. With a cohort of only 32 patients, the study may have been insufficiently powered to detect small or moderate effect sizes, and the observed negative findings may therefore represent type II errors. Accordingly, our results should be interpreted as indicating that no statistically significant associations were identified within the present cohort rather than demonstrating that such associations do not exist.

Nevertheless, the present study provides descriptive and exploratory data on a comparatively specific pediatric and adolescent trauma population. The findings may therefore contribute to hypothesis generation and provide preliminary estimates that can inform the design of future studies. Larger, preferably multicenter cohorts are required to provide adequate statistical power, enable more robust subgroup analyses, and determine whether the associations investigated in the present study can be confirmed or refuted. Future prospective studies with predefined hypotheses and appropriate sample size calculations would be particularly valuable for establishing more definitive conclusions.

Furthermore, long-term neurological, functional, and quality-of-life outcomes were not systematically available, restricting the assessment of recovery beyond hospital discharge. Although discharge disposition was documented, detailed follow-up data regarding neurocognitive development, physical functioning, and long-term rehabilitation outcomes could not be evaluated.

Despite these limitations, the present study provides valuable insights into the epidemiology, injury patterns, management, and short-term outcomes of pediatric polytrauma patients treated at a tertiary trauma center. The predominance of TBI, the frequent need for surgical intervention, and the occurrence of complications in a subset of patients underline the complexity of pediatric trauma care. The favorable in-hospital outcomes observed in this cohort, including the absence of mortality and the high proportion of patients discharged directly home, support the importance of specialized multidisciplinary management involving trauma surgeons, neurosurgeons, pediatric intensivists, orthopedic surgeons, and rehabilitation specialists.

Furthermore, the systematic assessment of injury severity using the ISS and regional MAIS scores provides a standardized framework for characterizing injury burden in pediatric polytrauma. Although no significant associations between head MAIS, ISS, and length of hospital stay were identified in the present cohort, these scoring systems remain valuable tools for injury classification, risk stratification, and comparison across trauma populations.

## Conclusion

Pediatric polytrauma remains a complex clinical condition that requires coordinated multidisciplinary management and specialized trauma care. In this single-center cohort, TBI was the predominant injury pattern, with traffic accidents representing the leading mechanism of injury. Although many patients sustained severe injuries requiring intensive care treatment and surgical intervention, short-term outcomes were generally favorable, with no in-hospital mortality and the majority of patients being discharged directly home. The predominance of cranial injuries highlights the central role of neurosurgical and neurocritical care expertise in the treatment of severely injured children. While no significant associations were identified between head MAIS, ISS, and length of hospital stay, severe TBI was frequently associated with complex clinical courses and the occurrence of complications. Thoracic, abdominal, pelvic, and extremity injuries further contributed to the overall trauma burden and often required multidisciplinary management. These findings emphasize the importance of specialized pediatric trauma systems, early multidisciplinary assessment, and individualized treatment strategies. Future multicenter studies with larger patient populations and long-term functional follow-up are needed to further clarify predictors of outcome and optimize the management of pediatric polytrauma patients.

Table 1. Baseline characteristics table, including demographics, Range of MAIS and ISS, as well as complications, duration of stay on intensive care units and discharge dispositions. It also includes information regarding GCS levels of the patients, showing that about 25% of them presented with a GCS lower than 8. Values are presented as absolute numbers and percentages.

**Table 1:** Baseline, injury and clinical characteristics of the study cohort

Characteristic	Total cohort (*N* = 32)
**Demographics**	
Age at admission, years¹	13.5 (8.8–16.0)
**Admission year**	
2023	12 (37.5%)
2024	10 (31.3%)
2025	10 (31.3%)
**Mechanism of injury**	
Traffic accident	24 (75.0%)
Fall	4 (12.5%)
Horse-riding fall	1 (3.1%)
Penetrating injury (knife)	1 (3.1%)
Trampoline injury	1 (3.1%)
Suicide attempt	1 (3.1%)
**Injury severity**	
ISS, median (IQR)	27 (20–34)
ISS, range	16–50
MAIS, median (IQR)	4 (4–5)
MAIS 3	3 (9.4%)
MAIS 4	17 (53.1%)
MAIS 5	12 (37.5%)
GCS, median (IQR)	14 (9–15)
GCS ≤ 8	7 (21.9%)
Intubation	8 (25.0%)
**Injury pattern by AIS body region²**	
Head/neck injury	22 (68.8%)
Facial injury	14 (43.8%)
Thoracic injury	10 (31.3%)
Abdominal injury	10 (31.3%)
Extremity/pelvic injury	9 (28.1%)
External injury	11 (34.4%)
**Treatment**	
Operative treatment	15 (46.9%)
No operative treatment	17 (53.1%)
**Hospital course³**	
ICU length of stay, days, median (IQR)	3 (1–5)
ICU length of stay, range	0–69
**Complications**	
Any documented complication	5 (15.6%)
No documented complication	27 (84.4%)
**Outcome**	
Good outcome	27 (84.4%)
Occasional headaches	2 (6.3%)
Post-traumatic headache	1 (3.1%)
Neurological disability⁴	1 (3.1%)
Psychiatric treatment	1 (3.1%)
In-hospital mortality	0 (0%)
**Discharge disposition**	
Home	27 (84,4%)
Rehabilitation	2 (6,3%)
Transfer to external hospital	3 (9,3%)

**Notes**

Data are presented as n (%) or median (interquartile range), unless otherwise indicated.

¹ Age is approximate because only admission year, not exact admission date, was provided.

² Patients may have injuries in multiple AIS body regions; percentages therefore do not sum to 100%.

³ Available variable is ICU length of stay, not total hospital length of stay.

⁴ Neurological disability: paralysis, wheelchair dependence and communication deficits.

Abbreviations: AIS, Abbreviated Injury Scale; GCS, Glasgow Coma Scale; ICU, intensive care unit; IQR, interquartile range; ISS, Injury Severity Score; MAIS, Maximum AIS.

## Data Availability

No datasets were generated or analysed during the current study.
